# Contrast‐Induced encephalopathy following diagnostic coronary angiography

**DOI:** 10.1002/ccr3.5624

**Published:** 2022-03-20

**Authors:** Hytham Rashid, Jonathan Brown, Emily Nix, Alan Fisher Covin

**Affiliations:** ^1^ University of Houston College of Medicine Houston USA; ^2^ HCA Houston Healthcare Kingwood USA; ^3^ 24780 Merit Health Wesley Hattiesburg USA

**Keywords:** cardiovascular disease, complication, contrast agents, coronary artery disease, diagnostic angiography

## Abstract

Contrast‐induced encephalopathy (CIE) is a rare, reversible complication of coronary angiography that can mimic acute strokes. This case illustrates the diagnostic challenges for a patient presenting with confusion following coronary angiography and raises awareness for CIE as diagnosis of exclusion.

## Graphical abstract

Contrast‐induced encephalopathy can mimic acute strokes warranting a high index of suspicion to prevent further contrast exposure to improve patient outcomes.

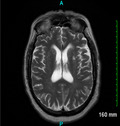

## 1 INTRODUCTION

CIE is a rare, acute, reversible, and self‐limiting neurologic complication of various angiographic procedures due to contrast exposure irrespective of the osmolarity or ionic state of the contrast agent.^1^, ^2^ Manifestations include visual disturbance, encephalopathy, motor, and sensory deficits, ophthalmoplegia, aphasia, and seizures.^2^ Symptoms typically begin within minutes to hours of contrast administration and resolve within one to two days.^2^ The pathophysiology may involve disruption of the blood–brain barrier leading to direct neurotoxicity and cerebral edema.^2^ Previous studies identified multiple risk factors for CIE including renal dysfunction, previous stroke, male gender, hypertension, and large volumes of contrast.^1^, ^3^ Recent advancements in coronary angiography reduced the complication rate of diagnostic procedures to less than 2% with a mortality less than 0.08%.^4^ Head CT and brain MRI are important diagnostic tests to exclude hemorrhage, thromboembolic processes, residual contrast enhancement, cerebral edema, or diffuse cortical hyper‐attenuation.^3^, ^5^ Treatment strategies vary from corticosteroids, intravenous fluids hydration, and close observation immediately following the procedure.^6^ Here, we present a case of delayed onset CIE as evident by altered mental status, aphasia, and tremors four days after coronary angiography.

## 2 CASE REPORT

**FIGURE 1:**
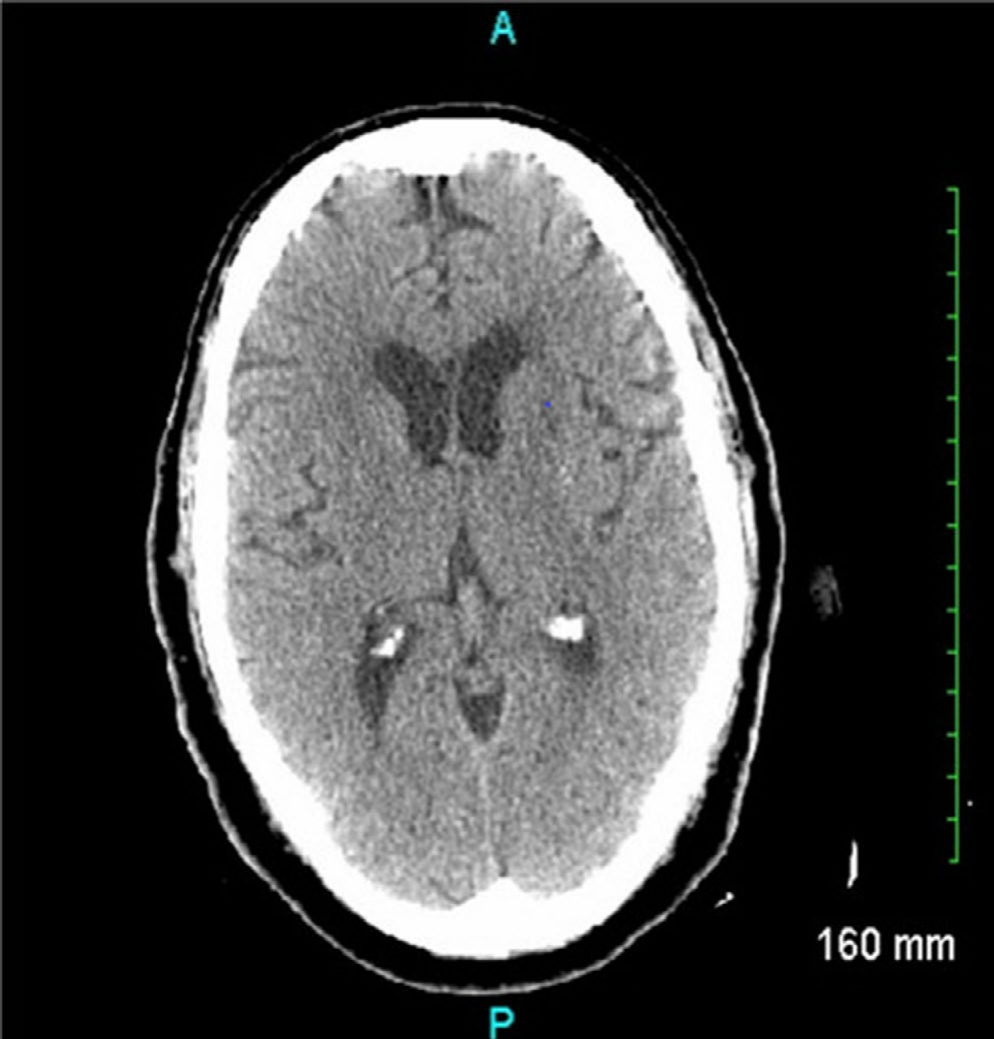
CT Head on admission. CT head scan without contrast showing no acute abnormalities

A 76‐year‐old Caucasian male patient presented to the emergency department (ED) with reported altered mental status by his family. The patient had been displaying confusion and aggressive behavior since earlier in the day. On arrival, the patient was afebrile with a blood pressure of 187/123 mmHg, a heart rate of 82 bpm, respiratory rate of 20 breaths/minute, and oxygen saturation of 96% on ambient air. Laboratory results were remarkable for a blood glucose of 202 mg/dL and a magnesium of 1.2 mg/dL. During bedside assessment, the patient adamantly stated that there was “nothing wrong” with him; however, he was unable to verbalize full sentences and did not follow commands on questioning. The National Institute of Health Stroke Scale score was 6. A new, slow, coarse, bilateral wrist tremor was also noted at rest, which worsened with limb manipulation, and was more pronounced on the right. Computed tomography (CT) of the head without contrast showed no acute abnormalities (Figure 1). Neurology was consulted and concluded that the abnormal upper extremity movement was likely due to a rubral tremor.

**FIGURE 2:**
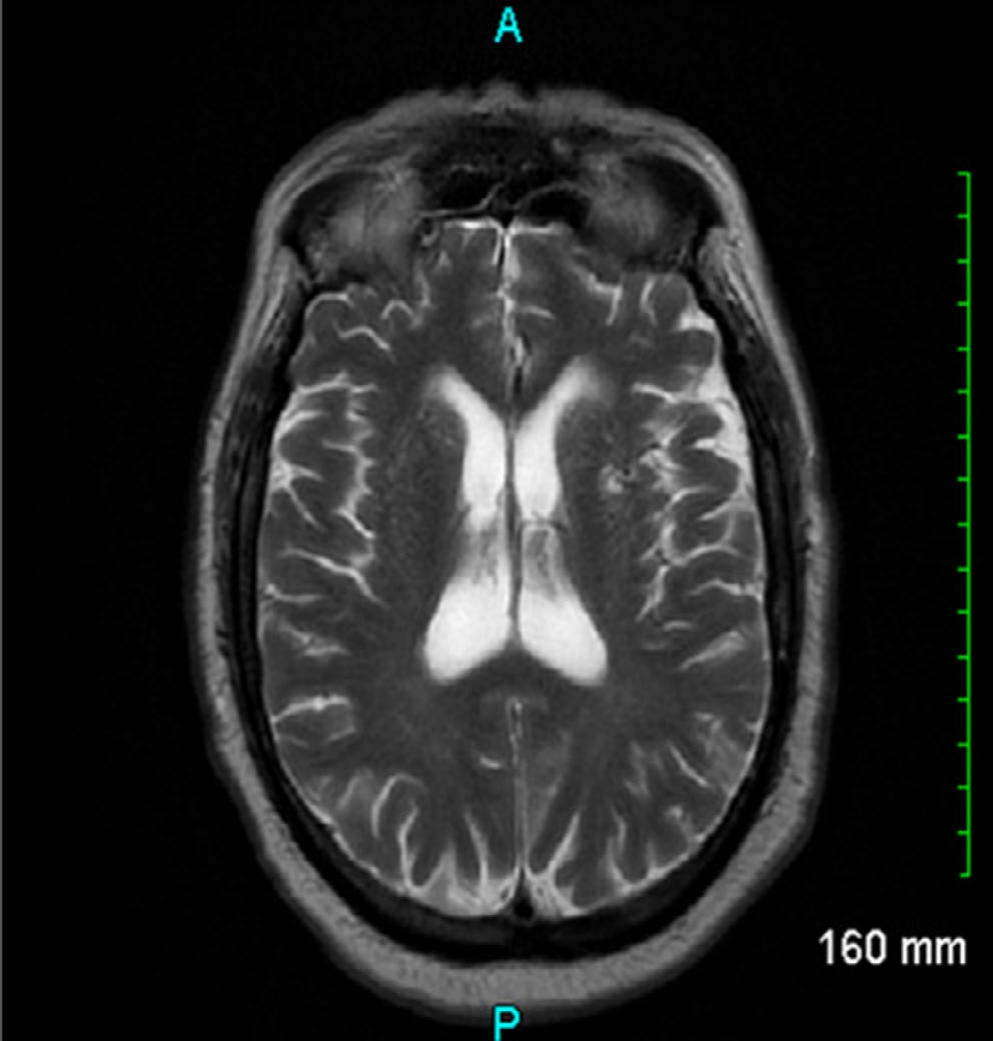
MRI Brain on second day. MRI of the brain without contrast showing atrophy with white matter changes, however, no acute abnormality

**FIGURE 3:**
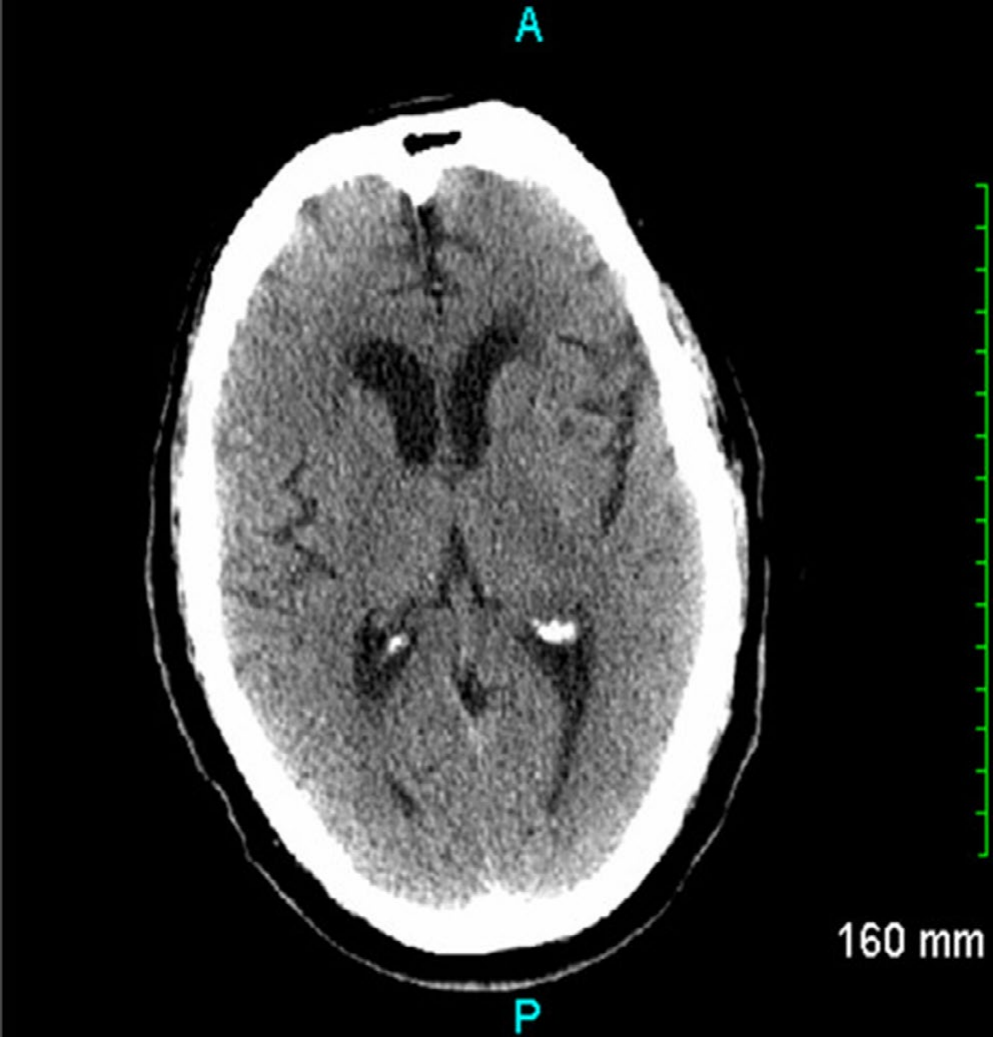
CT Head on sixth day. Repeat CT head without contrast showed no acute abnormalities

The patient's past medical history was significant for non‐insulin‐dependent diabetes mellitus, hypertension, hyperlipidemia, coronary artery disease (CAD) with a history of seven cardiac catheterizations and placement of four drug‐eluting stents. His wife denied any history of alcohol, tobacco, or illicit drug use. Family history was non‐contributory. She also denied any recent travel or exposure to sick contacts or toxins. She did, however, state that the patient had experienced intermittent chest discomfort one month prior for which he underwent nuclear stress testing that showed moderate probability reversible apical lateral ischemia. Four days before his arrival to the ED, the patient had a diagnostic coronary angiography performed which revealed a left ventricular ejection fraction >60% and moderate to severe, diffuse multivessel CAD within the left anterior descending artery (in‐stent restenosis <50%), left circumflex artery, and a dominant right coronary artery. The patient was referred to cardiothoracic surgery for evaluation for coronary artery bypass graft (CABG) and prescribed daily oral doses of aspirin 81 mg, atenolol 100 mg, lisinopril 20 mg, clopidogrel 75 mg, rosuvastatin 5 mg, and spironolactone 25 mg. His wife reported that he did not take at least two doses of clopidogrel. Differential diagnosis for this patient included acute ischemic stroke, subarachnoid hemorrhage, reperfusion syndrome, hypertensive emergency, and an evolving cerebral infarction. The patient was admitted to the hospital for continued monitoring, and cardiology was consulted for further evaluation. An electrocardiogram showed an accelerated junctional rhythm, left axis deviation, and inferior infarct, age undetermined. CT angiography of the head and neck showed no stenosis of the carotid or vertebral arteries. Further testing including urine analysis, urine drug screen, blood cultures, cerebrospinal fluid analysis, coagulation studies, and serum heavy metal concentrations were all unremarkable. A magnetic resonance imaging (MRI) of the brain without contrast showed atrophy with white matter changes, however, no acute abnormality (Figure 2). A transthoracic echocardiogram showed normal systolic function with moderate left ventricular hypertrophy and trace mitral, tricuspid, and pulmonic regurgitation. On the sixth day of admission, the patient developed dystonic movements. A repeat head CT was performed and continued to show no acute abnormalities (Figure 3). Neurology ordered an electroencephalogram, which showed no epileptiform activity.

The patient's hypertension resolved with hydralazine; his magnesium was repleted; and his blood glucose was treated with subcutaneous insulin. Conversely, his cognitive status worsened to where he became combative and required restraints. The patient began to display increased lucidity on Day 10, and on Day 16, he showed persistent cognitive improvement and the rubral tremors resolved without pharmacologic therapy. After excluding other possible etiologies, contrast‐induced encephalopathy (CIE) was concluded as the final diagnosis. At a 3‐month follow‐up with his cardiologist, the patient denied any further symptom recurrence, reported medication compliance, and declined any further evaluation from cardiothoracic surgery for a possible CABG.

## 3 DISCUSSION

CIE is a transient neurologic complication following coronary angiography that presents with various clinical effects including motor and sensory deficits, cortical blindness, aphasia, and seizures.^2^ CT findings may be normal, or present with cortical/subcortical contrast enhancement and vasogenic edema on MRI.^2^ In our case, a non‐contrast CT and brain MRI performed on admission showed no intracranial pathological findings. Although large volumes of contrast are a known risk factor, minimal administration of contrast has also been reported to cause CIE.^7^ Male gender, diabetes mellitus, and hypertension were important risk factors for CIE in our patient. Hypertension can impair the blood–brain barrier and serves as a predisposing factor for contrast extravasation.^8^ Of note, our patient did not have any prior adverse reaction to iodinated contrast. A previous report showed that re‐exposure to contrast media may precipitate a recurrence of CIE despite premedication with corticosteroids and antihistamines.^2^, ^9^

Rubral tremors with normal brain imaging after cardiac catheterization have been demonstrated in prior cases.^10^, ^11^ The initial cardiac catheterization in this patient followed by acute onset of the tremor demonstrated a strong cause‐and‐effect relationship, as the tremor improved in conjunction with the patient's mental status. Rubral tremor presents as involuntary shaking during rest that is exacerbated by voluntary movement. It is a specific clinical syndrome that may involve lesions of the superior cerebellar peduncle, midbrain tegmentum, or posterior part of the thalamus.^12^ In our case, the patient's rubral tremor required neurologic consultation and resolved without requiring pharmacotherapy.

The prognosis of CIE is excellent with supportive management.^3^ However, recovery time in our patient was prolonged compared to a prior retrospective analysis of 9 patients with CIE, which showed a median time to resolution of symptoms was 14 h.^13^

## 4 CONCLUSION

CIE is a diagnosis of exclusion, occurring as a complication of contrast exposure with variable symptom onset and duration, where hyperosmolar contrast may disrupt the blood–brain barrier. Radiologic findings may mimic a subarachnoid hemorrhage or may be unremarkable, as in this case. Despite its challenging diagnosis, CIE carries a good prognosis with supportive care focused primarily on adequate hydration.

## CONFLICTS OF INTEREST

The authors of this manuscript have no conflicts of interest to report.

## AUTHOR CONTRIBUTIONS

Hytham Rashid cared for the patient and wrote the introduction and case report. Dr. Jonathan Brown contributed to the discussion and revised the manuscript. Dr. Emily Nix directly cared for the patient and reviewed the manuscript. Dr. Alan Covin directly cared for the patient and reviewed the manuscript.

## ETHICAL APPROVAL

This manuscript was approved by the local Institutional Review Board (IRB) of HCA Houston Kingwood and granted IRB exemption.

## CONSENT

Written informed consent was obtained from the patient to publish this report in accordance with the journal's patient consent policy.

## Data Availability

Data are available upon reasonable request.

## References

[1] Chu YT , Lee KP , Chen CH , et al. Contrast‐induced encephalopathy after endovascular thrombectomy for acute ischemic stroke. Stroke. 2020;51(12):3756‐3759. 33121385 10.1161/STROKEAHA.120.031518

[2] Spina R , Simon N , Markus R , Muller DWM , Kathir K . Contrast‐induced encephalopathy following cardiac catheterization. Catheter Cardiovasc Interv. 2017;90(2):257‐268. doi:10.1002/ccd.26871 27896904

[3] Yu J , Dangas G . Commentary: new insights into the risk factors of contrast‐induced encephalopathy. J Endovasc Ther. 2011;18(4):545‐546. doi:10.1583/11-3476C.1 21861746

[4] Tavakol M , Ashraf S , Brener SJ . Risks and complications of coronary angiography: a comprehensive review. Glob J Health Sci. 2012;4(1):65‐93. 22980117 10.5539/gjhs.v4n1p65 PMC4777042

[5] Allison C , Sharma V , Park J , Schirmer CM , Zand R . Contrast‐induced encephalopathy after cerebral angiogram: a case series and review of literature. Case Rep Neurol. 2021;13(2):405‐413. 34248578 10.1159/000516062 PMC8255655

[6] Leong S , Fanning NF . Persistent neurological deficit from iodinated contrast encephalopathy following intracranial aneurysm coiling. a case report and review of the literature. Interv Neuroradiol. 2012;18(1):33‐41. 22440599 10.1177/159101991201800105 PMC3312087

[7] Potsi S , Chourmouzi D , Moumtzouoglou A , Nikiforaki A , Gkouvas K , Drevelegas A . Transient contrast encephalopathy after carotid angiography mimicking diffuse subarachnoid haemorrhage. Neurol Sci. 2012;33(2):445‐448. 21927883 10.1007/s10072-011-0765-3

[8] Harnish PP , Hagberg DJ . Contrast media‐induced blood‐brain barrier damage damage. potentiation by hypertension. Invest Radiol. 1988;23(6):463‐465. 3403206 10.1097/00004424-198806000-00009

[9] Spina R , Simon N , Markus R , Muller DW , Kathir K . Recurrent contrast‐induced encephalopathy following coronary angiography. Intern Med J. 2017;47(2):221‐224. 28201864 10.1111/imj.13321

[10] Dattani A , Au L , Tay KH , Davey P . Contrast‐induced encephalopathy following coronary angiography with no radiological features: a case report and literature review. Cardiology. 2018;139(3):197‐201. 29402812 10.1159/000486636

[11] Restrepo L , Quiroga‐Chand A , Tomatore C , Balish M . "Rubral" tremor after cardiac catheterization: report of 2 cases. Tex Heart Inst J. 2005;32(3):427‐429. 16392236 PMC1336726

[12] Berkovic SF , Bladin PF . Rubral tremor: clinical features and treatment of three cases. Clin Exp Neurol. 1984;20:119‐128. 6568936

[13] Kocabay G , Karabay CY , Kalayci A , et al. Contrast‐induced neurotoxicity after coronary angiography. Herz. 2014;39(4):522‐527. 23846826 10.1007/s00059-013-3871-6

